# Hepatic Safety of Rilpivirine/Emtricitabine/Tenofovir Disoproxil Fumarate Fixed-Dose Single-Tablet Regimen in HIV-Infected Patients with Active Hepatitis C Virus Infection: The hEPAtic Study

**DOI:** 10.1371/journal.pone.0155842

**Published:** 2016-05-19

**Authors:** Karin Neukam, Nuria Espinosa, Antonio Collado, Marcial Delgado-Fernández, Patricia Jiménez-Aguilar, Antonio Rivero-Juárez, Victor Hontañón-Antoñana, Ana Gómez-Berrocal, Josefa Ruiz-Morales, Dolores Merino, Ana Carrero, Francisco Téllez, María José Ríos, José Hernández-Quero, María de Lagarde-Sebastián, Inés Pérez-Camacho, Francisco Vera-Méndez, Juan Macías, Juan A. Pineda, on behalf of the hEPAtic Study Group

**Affiliations:** 1 Unit of Infectious Diseases and Microbiology, Hospital Universitario de Valme, Seville, Spain; 2 Service of Infectious Diseases, Hospital Universitario Virgen del Rocío, Seville, Spain; 3 Unit of Infectious Diseases, Hospital Torrecárdenas, Almeria, Spain; 4 Unit of Infectious Diseases, Hospital Regional de Málaga. Malaga, Spain; 5 Unit of Infectious Diseases, Hospital Universitario Puerto Real, Puerto Real, Spain; 6 Unit of Infectious Diseases, Hospital Universitario Reina Sofía, Instituto de Investigación Biomédica de Córdoba (IMIBIC), Cordoba, Spain; 7 HIV Unit, Service of Internal Medicine, Hospital Universitario La Paz/IdiPAZ, Madrid, Spain; 8 Service of Internal/Infectious Medicine, Hospital Universitario de la Princesa, Madrid, Spain; 9 Unit of Infectious Diseases, Hospital Universitario Virgen de la Victoria, Malaga, Spain; 10 Unit of Infectious Diseases, Complejo Hospitalario de Huelva, Huelva, Spain; 11 Unit of Infectious Diseases/HIV, Hospital General Universitario Gregorio Marañón, Madrid, Spain; 12 Unit of Infectious Diseases, Hospital La Línea, AGS Campo de Gibraltar, La Linea de la Concepcion, Spain; 13 Unit of Infectious Diseases, Hospital Virgen Macarena, Seville, Spain; 14 Unit of Infectious Diseases, Hospital Universitario San Cecilio, Granada, Spain; 15 HIV Unit. Hospital Universitario 12 de Octubre, Madrid, Spain; 16 Tropical Medicine Unit, Hospital Poniente, El Ejido, Spain; 17 Infectious Medicine Section, Hospital Universitario Santa Lucia, Cartagena, Spain; Harvard Medical School, UNITED STATES

## Abstract

**Objectives:**

The aim of this study was to evaluate the frequency of transaminase elevations (TE) and total bilirubin elevations (TBE) during the first year of therapy with a single tablet regimen including RPV/FTC/TDF (EPA) in HIV/hepatitis C virus (HCV)-coinfected subjects in clinical practice.

**Methods:**

In a retrospective analysis, HIV/HCV-coinfected subjects who started EPA at 17 centres throughout Spain were included as cases. Subjects who started an antiretroviral therapy (ART) other than EPA during the study period at the same hospitals were randomly selected as controls in a 1:2 ratio. Primary outcome variables were grade (G) 3–4 TE and G4 TBE.

**Results:**

Of the 519 subjects included, 173 individuals started EPA. Nine (5.2%) subjects of the EPA group and 49 (14.2%) controls were naïve to ART. The median (Q1–Q3) follow-up was 11.2 (9.7–13.9) months. TE was observed in 2 [1.2%; 95% confidence interval (CI): 0.14%–4.1%] subjects receiving EPA and 11 (3.2%; 95%CI: 1.6%–5.6%) controls (p = 0.136), all events were G3. No patient discontinued ART due to TE. One (0.6%; 95%CI: 0.01%–3.1%) subject on EPA and 8 (2.3%; 95%CI: 1%–4.5%) subjects in the control group developed TBE (p = 0.141), without developing any other hepatic event during follow-up. Three (2.3%) subjects with cirrhosis versus 10 (3.1%) without cirrhosis showed G3-4 TE (p = 0.451).

**Conclusion:**

The frequency of severe liver toxicity in HIV/HCV-coinfected subjects receiving EPA under real-life conditions is very low, TE were generally mild and did not lead to drug discontinuation. All these data suggest that EPA can be safely used in this particular subpopulation.

## Introduction

Antiretroviral therapy (ART) may lead to hepatotoxic events, such as liver enzyme elevations, acute liver failure and death [1]. These events occur more frequently in HIV/HCV-coinfected subjects as compared to HIV monoinfected patients [2–6]. Liver toxicity at the beginning of the ART era was relatively common in HIV/HVC-coinfected subjects, with high risk of severe and eventually fatal hepatic events. Among non-nucleoside reverse transcriptase inhibitors (NNRTI), nevirapine demonstrated a higher risk of hepatotoxicity as compared to efavirenz (EFV) [7] and among protease inhibitors (PIs), tipranavir and higher doses of ritonavir were more toxic than other members of this drug family [8]. However, most of these drugs are not used nowadays. Conversely, newer antiretroviral drugs, such as boosted atazanavir (ATV) or darunavir (DRV), as well as integrase inhibitors, have been associated to a lower rate of toxic liver events in the clinical practice [2,6,9].

Rilpivirine is a newer NNRTI that is given coformulated with tenofovir and emtricitabine as a single tablet regimen (EPA) or as single drug along with other antiretroviral drugs. In a pooled analysis of the two pivotal phase III clinical trials on RPV, the hepatic safety profile in subjects with chronic viral hepatitis appeared somewhat poorer as compared to HIV-monoinfected subjects [10]. However, due to the relatively low frequency of HIV/HCV-coinfected patients in these studies, these figures need confirmation and additionally, these studies did not evaluate a single tablet regimen (STR). The STaR and SPIRIT clinical trials did evaluate these drugs in a STR, however, likely due to the low number of patients with HCV and/or HBV coinfection, no data on hepatic safety in this subpopulation is available [11,12]. Finally, no data on this topic based on routine clinical data are available. Consequently, studies on the hepatic safety of EPA, particularly those based on real-life experience, are warranted.

The aim of the present study was to evaluate the frequency of severe hepatic toxicity, defined as grade 3 or 4 transaminase elevations (TE) or grade 4 total bilirubin elevations (TBE), during the first 48 weeks of EPA in HIV-infected subject with chronic HCV infection.

## Patients and Methods

### Study design and study population

In a retrospective case-control study, all patients consecutively seen at the Infectious Diseases Units of 17 Spanish hospitals between November 2012 and February 2014, were selected disregarding the presence or severity of hepatic toxicities if they fulfilled the following criteria: i) Older than 18 years; ii) HIV-1 infection, as diagnosed on the basis of the presence of serum HIV antibodies detected by EIA and western-blot; iii) Chronic HCV infection as confirmed by detectable plasma HCV-RNA; iv) Starting a new antiretroviral drug regimen; v) Exposure to study drugs for at least one week; vi) Clinical visits and blood tests available at baseline and, at least, after 12, 24 and 48 weeks thereafter, unless treatment was discontinued for any reason. In those patients who received therapy against HCV-infection, follow-up was stopped at the moment of anti-HCV treatment initiation. Likewise, treatment interruption due to any reason required immediate stop of follow-up resulting to drug exposure being reflected by the time of follow-up.

### ART Regimens

Out of the overall population included in the database, patients naïve for RPV were selected in a 1:2 (case/control) ratio according to the following regimens: i) Cases (EPA group): All patients who had started EPA during the study period and ii) Controls: Subjects who initiated any ART that did not include RPV during the study period. The control group was randomly selected out of all the patients with ART changes or initiation of one or more new antiretroviral drugs in the participant hospitals.

### Definition of liver enzyme and bilirubin elevations

Grade 3 TE were defined as elevations of alanine-aminotransferase (ALT) or aspartate-aminotransferase (AST) between 5 and 10 times above the upper level of normality (ULN) among patients who had normal baseline levels. Grade 4 TE were defined as ALT or AST increases greater than 10 times the ULN. In patients with elevated baseline ALT or AST levels, 3.5- to 5-fold increase from baseline levels were considered grade 3 TE and greater than 5-fold elevations were defined as grade 4 TE. Grade 1 and grade 2 TE was analysed in those patients with baseline ALT and AST values below the ULN and were defined as ALT or AST levels 1.25 to 2.5-fold and 2.5 to 5-fold above the ULN, respectively. Grade 4 TBE were defined as increases of total bilirubin equal or greater than 5 mg/dL. Likewise, grade 1, grade 2 and grade 3 TBE were considered when total bilirubin levels were ≥1.1 mg/dL to <1.6 mg/dL, ≥1.6 mg/dL to <2.6 mg/dL and ≥2.6 mg/dL to <5 mg/dL, respectively [13].

### Liver fibrosis assessment

Baseline advanced fibrosis and cirrhosis was diagnosed when stage 3 (F3) or stage 4 (F4) according to the Scheuer index was detected in a liver biopsy [14]. If biopsy was not available, transient elastography cut-off values of ≥9.5 kPa for advanced fibrosis and ≥14.6 kPa for cirrhosis were applied [15,16]. In patients without liver biopsy or liver stiffness measurement, advanced fibrosis was excluded when baseline FIB-4 index was ≤1.45 and diagnosed when baseline FIB-4 values were ≥3.25 [17]. Both liver biopsy and transient elastography were considered valid if obtained within six months before or after ART initiation. In the case of F4, a 12 month-period before ART initiation was allowed.

### Statistical analysis

The frequency of subjects who present grade 3 or 4 TE and/or grade 4 TBE, as well as of those who discontinue ART due to adverse events, were assessed and the 95% confidence interval (CI) was calculated. Comparisons of continuous variables were carried out by means of the Student’s t-test or the Mann-Whitney U-test, when applicable. To compare categorical variables, the χ2 test or the Fisher’s exact test were used. In order to compare the CD4 cell counts and the HIV viral loads at baseline and the end of follow-up, the Wilcoxon Signed Rank test and the McNemar test were applied, respectively. A univariate analysis was carried out to identify baseline factors associated with hepatic toxicity. Subsequently, a multivariate logistic regression analysis was applied, adjusting for age, sex, as well as those factors that were associated with a p<0.2 in the univariate analysis. Statistical analysis and control randomization was performed using the SPSS statistical software package release 22.0 (IBM, Chicago, IL, USA) and R i386 3.0.1 (The R Foundation for Statistical Computing, Boston, USA).

### Ethical aspects

The study was designed and performed according to the Helsinki declaration and was approved by the Autonomic Ethics Committees of Andalusia (Comité Coordinador de Ética de la Investigación Biomédica de Andalucía, CCEIBA). Patients gave their written informed consent.

## Results

### Characteristics of the patients

One hundred and seventy-three patients who initiated EPA were included in this study. Accordingly, 346 controls were randomly selected. Median (IQR) age was 47.6 (43.7–50.8) years and 427 (82.3%) were male. The demographic and baseline characteristics of the study population are listed in Table 1. Table 2 lists the reasons for ART initiations by treatment group.

**Table 1:** Baseline characteristics of the study population (n = 519). EPA: RPV/TDF/FTC group.

Characteristic	Study groups		*p*
	EPA group	Control group	
	n = 173	n = 346	
**Male gender, n (%)**	146 (84.4)	281 (81.2)	0.371
Age*, years	47.9 (44.5–51)	47 (43.2–50.7)	0.103
**Intravenous drug use, n (%)**	138 (79.8)	279 (80.6)	0.815
**Alcohol intake >50 g/day, n (%)**	19 (11)	50 (14.5)	0.273
**HBsAg(+), n (%)**	5 (3.1)	4 (1.2)	0.129
**CDC clinical category C, n (%)**	51 (29.5)	127 (36.7)	0.102
**HIV RNA >1000 copies/mL**^1^**, n (%)**	26 (15)	108 (31)	<0.001
CD4 cell counts*, cells/mL	515 (332–764)	398 (240–589)	<0.001
Plasma HCV RNA*, log_**10**_ **IU/mL**	6.16 (5.67–6.78)	6.19 (5.72–6.63)	0.482
**HCV genotype**^2^**, n (%)**			0.849
** 1**	108 (62.5)	214 (62.8)	
** 2**	1 (0.6)	2 (0.6)	
** 3**	26 (15.8)	50 (14.7)	
** 4**	30 (18.2)	74 (21.7)	
** 6**	0	1 (0.3)	
**Treatment naïve for anti-HCV**	99 (57.2)	246 (71.1)	0.002
**therapy, n (%)**			
ALT*, IU/mL	48 (33–69)	47 (31.8–75.3)	0.795
AST*, IU/mL	48 (33–69)	47 (31–70.3)	0.535
Total bilirubin*, mg/dL	0.52 (0.4–0.93)	0.6 (0.41–0.9)	0.146
**Advanced fibrosis**^3^**, n (%)**	61 (37.9)	145 (46.6)	0.07
**Cirrhosis**^4^**, n (%)**	42 (26.6)	91 (30.2)	0.413

*Median (Q1-Q3).

1: determined in those patients with detectable baseline HIV RNA (46 cases and 191 controls)

2: available in 165 cases and 341 controls

3: available in 161 cases and 311 controls

4: available in 158 cases and 301 controls.

**Table 2:** Reasons for initiation of antiretroviral therapy (ART) within the RPV/TDF/FTC group (EPA) and the control group; overall p <0.001.

Reason	EPA group	Control group
	n = 173	n = 346
**Naïve for ART**	9 (5.2)	49 (14.2)
**Reinitiation after therapy discontinuation**	10 (5.8)	37 (10.7)
**Simplification**	49 (28.3)	50 (14.5)
**Virological failure**	10 (5.8)	49 (14.2)
**Adverse events**	69 (39.9)	98 (28.3)
**Interactions with future anti-HCV treatment**	9 (5.2)	28 (8.1)
**Interactions with other drugs**	3 (1.7)	12 (3.5)
**Intensification**	0 (0)	5 (1.4)
**Other**	14 (8.1)	18 (5.2)

### ART regimens in the control group

Triple therapy with a NRTI backbone among the controls was mainly based on TDF/FTC, which was administered to 165 (47.7%) patients. NRTI-sparing regimens were administered to 92 (26.6%) individuals and abacavir (ABC)/ lamivudine (3TC) were provided to 59 (17.1%) subjects. A total of 475 drug initiations were registered in the control group. The newly introduced drugs are summarized in Table 3.

**Table 3:** Newly introduced antiretroviral therapy (ART) in the control group (n = 346).

Antiretroviral drug	Initiated ART,
	n (%)
**Nucleoside analogue reverse transcriptase inhibitors**	
**Tenofovir/emtricitabine**	75 (21.7)
**Abacavir/lamivudine**	43 (12.4)
**Other NRTI combinations**	38 (11)
**Ritonavir(r)-boosted protease inhibitors**	
**Lopinavir/r**	15 (4.3)
**Atazanavir/r**	48 (13.9)
**Darunavir/r**	114 (32.9)
**Non-nucleoside analogue reverse transcriptase inhibitors**	
**Efavirenz**	33 (9.5)
**Nevirapine**	10 (2.9)
**Etravirine**	30 (8.7)
**Integrase inhibitors**	
**Raltegravir**	45 (13)
**Entry inhibitors**	
**Maraviroc**	24 (6.9)

### Follow-up

**Fig 1:**
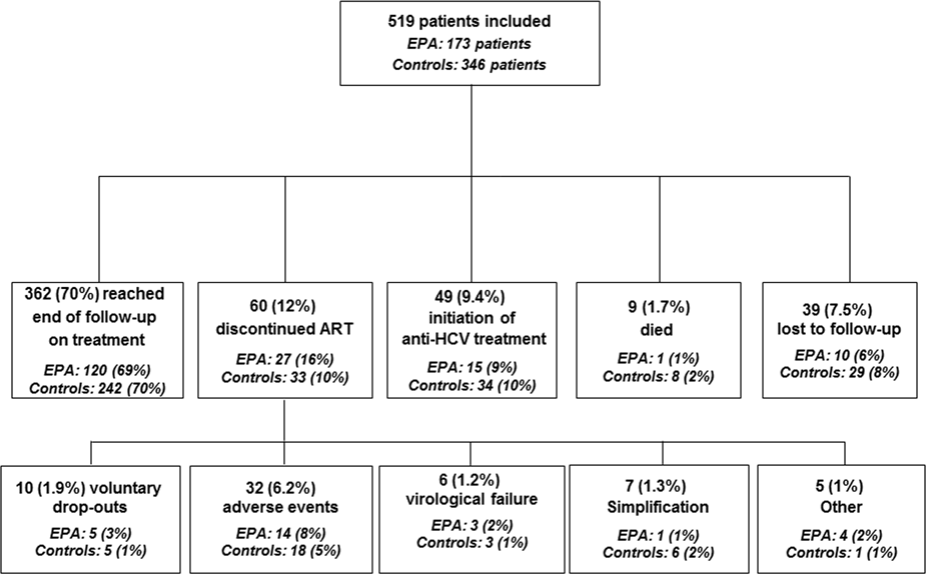
Patient disposition and treatment outcome according to study group. EPA: RPV/TDF/FTC group. None of the outcomes showed statistically significant differences between the two groups.

The median (IQR) follow-up of the study patients was 11.2 (6.2–12.4) months for the EPA group and 11.2 (7.8–12.5) months for the control group (p = 0.856), 362 (70%) subjects reached the scheduled end of follow-up. The numbers of patients who did not reach week 12 of therapy were 8 (4.6%) for the EPA group and 27 (7.8%) for the control group (p = 0.173). Therapy against HCV infection was initiated after 4.9 (3–9.7) months in the EPA group and 3.8 (2.5–6.8) months (p = 0.269). The detailed outcome for the two study populations is shown in Fig 1. The number of patients with plasma HIV RNA below 50 copies/mL at baseline versus after 12 months of follow-up was 133 (76.9%) versus 91 (89.2%; p = 0.263) among the patients who received EPA and 170 (49.1%) versus 169 (84.5%; p<0.001) in the control group (p = 0.173), respectively. Median (IQR) CD4 cell counts at baseline versus the end of follow-up were 515 (332–764) cells/mL versus 606 (362–819) cells/mL (p = 0.092) in the EPA group and 398 (240–589) cells/mL versus 465 (295–696) cells/mL (p<0001) among the controls.

### Liver safety

Grade 3 TE was observed in 2 (1.2%; 95%CI: 0.14%-4.1%) individuals receiving EPA and in 11 (3.2%; 95%CI: 1.6%-5.6%) patients of the control group (p = 0.136). All grades of TE according to the study group are summed up in Table 4. No episode of grade 4 TE during the study period was detected. In the EPA group, grade 3 TE episodes were observed after 24.4 and 25.6 weeks of therapy, respectively. In the control group, the time (IQR) to grade 3 TE was 16.4 (13–37.8) weeks. Among the subgroup of patients who initiated the new ART due to simplification, adverse events to prior ART or due to interactions, 2 (1.7%) patients who received EPA and 6 (3.8%) subjects of the control group showed grade 3 TE (p = 0.251).

**Table 4:** Frequency of different grades of transaminase elevations and total bilirubin elevations according to study group.

Event	Control group	EPA group	*p*
	n = 346	n = 173	
**Transaminase elevations**			
Grade 1*	51 (34)	28 (27.3)	0.621
Grade 2*	20 (13.8)	4 (6)	0.095
** Grade 3**	11 (3.2)	2 (1.2)	0.136
** Grade 4**	0	0	-
**Total bilirubin elevations**			
** Grade 1**	28 (9.9)	17 (12)	0.504
** Grade 2**	22 (7)	7 (4.4)	0.265
** Grade 3**	26 (7.9)	3 (1.8)	0.007
** Grade 4**	8 (2.3)	1 (0.6)	0.141

*Determined in the subpopulation who showed AST and ALT values below the upper limit of normality at baseline.

Grade 4 TBE were identified in 1 (0.6%; 95%CI: 0.01%-3.1%) patients on EPA and in 8 (2.3%; 95%CI: 1%-4.5%; p = 0.141) subjects of the control group. Of the latter, 7 patients received ritonavir-boosted atazanavir as part of their ART regimen. Other TBE grades according to study group are shown in Table 4. No patient discontinued ART due to liver toxicity and none of those individuals who developed grade 3 or 4 TE or grade 4 TBE developed other hepatic events during the follow-up.

Fourteen (8%; 95%CI: 4.4%-13%) of the EPA group and 18 (5.2%; 95%CI: 3.1%-8.1%) of the control group discontinued therapy due to any adverse event. Hepatic decompensations during the study period were observed in one (0.6%; 95%CI: 0.001%-3.2%) patient who received EPA and in 6 (1.7%; 95%CI: 0.6%-3.7%) subjects from the control group. The patient receiving EPA presented hepatic encephalopathy and portal hypertensive gastrointestinal bleeding. The episodes observed within the control group were: hepatic encephalopathy (5 patients), ascites (two patients) and portal hypertensive gastrointestinal bleeding (one patient). One (0.6%) patient of the EPA group and 8 (2.3%) patients of the control group died during the period of follow-up. Death due to hepatic events was observed in 1 (0.6%) patient of the EPA group and 2 (0.2%) patients of the control group (p = 0.741).

**Fig 2:**
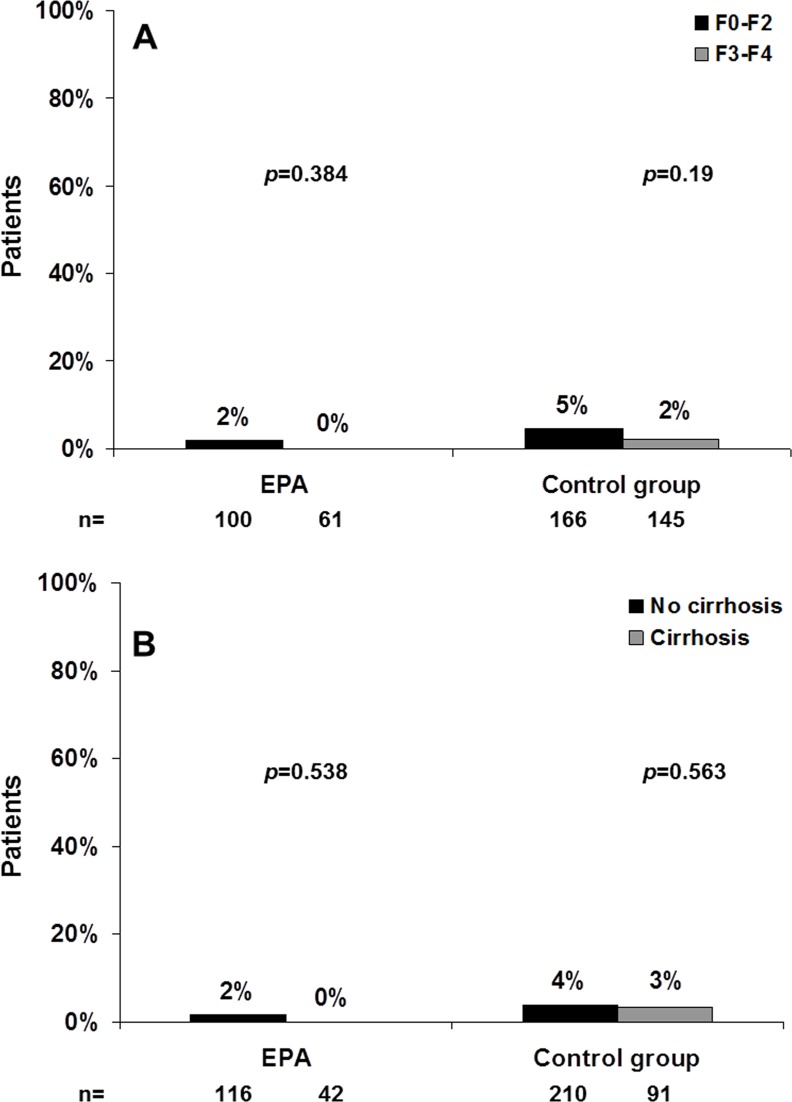
**Grade 3 transaminase elevations (TE) according to the presence of advanced fibrosis (2A) or cirrhosis (2B) at baseline by treatment group.** EPA: RPV/TDF/FTC group.

A total of 3 (1.5%) subjects with advanced fibrosis (F≥3) versus 10 (3.8%) without advanced fibrosis (F<3) showed grade 3 or 4 TE (p = 0.129). The corresponding figures for subjects with and without cirrhosis were 3 (2.3%) subjects and 10 (3.1%) patients, respectively (p = 0.451). The proportions of severe TE according to liver damage and within the study groups are shown in Fig 2.

### Predictors of grade 3 or 4 TE and grade 4 TBE

In the multivariate analysis, no predictors of grade 3 or 4 TE could be identified (Table 5). The initiation of ritonavir-boosted atazanavir was the only factor independently associated with grade 4 TBE (Table 6).

**Table 5:** Predictors of grade 3 or 4 transaminase elevations (TE).

	n	Grade 3 or 4	*p*	AOR	*p*
		TE, n (%)	uni-variate	(95% confidence interval)	multi-variate
Age*^1^					
** <47.6 years**	259	3 (3.1)	0.360	0.997	0.949
** ≥47.6 years**	260	5 (5.2)		(0.913–1.088)	
**Sex**					
** Male**	427	12 (2.8)	0.297	2.792	0.33
** Female**	92	1 (1.1)		(0.354–22.04)	
**Alcohol intake**					
** <50 g/day**	450	10 (2.2)	0.243		
** ≥50 g/day**	69	3 (4.3)			
ALT levels*					
** ≥40 IU/mL**	329	10 (3)	0.236		
** <40 IU/mL**	190	3 (1.6)			
**CDC category**					
** C**	178	4 (2.2)	0.522		
** A or B**	341	9 (2.6)			
**Undetectable HIV**					
**RNA**					
** Yes**	282	6 (2.1)	0.549		
** No**	237	7 (3)			
**Advanced fibrosis**					
** Yes**	206	3 (1.5)	0.129	3.312	0.087
** No**	266	10 (3.8)		(0.84–13.049)	
CD4 cell count*^2^					
** <350 cells/mL**	186	6 (1.8)	0.148	0.999	0.405
** ≥350 cells/mL**	327	7 (3.8)		(0.997–1.001)	
**EPA treatment**					
** Yes**	173	2 (1.2)	0.136	0.337	0.169
** No**	346	11 (3.2)		(0.071–1.589)	
**Start of a NRTI**					
** Yes**	194	6 (3.1)	0.348		
** No**	325	7 (2.2)			
**Start of a PI/r**					
** Yes**	177	6 (3.4)	0.258		
** No**	342	7 (2)			
**Start of raltegravir**					
** Yes**	45	1 (2.2)	0.687		
** No**	474	12 (2.5)			
**Start of a maraviroc**					
** Yes**	24	1 (4.2)	0.464		
** No**	495	12 (2.4)			

^1^Categorized by median

^2^available in 513 patients

*entered as continuous variable in the multivariate analysis. AOR: adjusted odd´s ratio; EPA: RPV/TDF/FTC; PI/r: ritonavir-boosted protease inhibitor; NRTI: nucleos(t)ide reverse transcriptase inhibitors.

**Table 6:** Predictors of grade 4 total bilirubin elevations (TBE).

	n	Grade 4	*p*	AOR	*p*
		TBE, n(%)	uni-variate	(95% confidence interval)	multi-variate
Age*^1^					
** ≥47.6 years**	260	3 (1.2)	0.25	0.988	0.832
** <47.6 years**	259	6 (2.3)		(0.881–1.107)	
**Sex**					
** Male**	427	8 (1.9)	0.506	1.874	0.561
** Female**	92	1 (1.1)		(0.223–15.75)	
**Alcohol intake**					
** <50 g/day**	450	9 (2)	0.274		
** ≥50 g/day**	69	0 (0)			
ALT levels*					
** ≥40 IU/mL**	329	7 (2.1)	0.366		
** <40 IU/mL**	190	2 (1.1)			
**CDC category**					
** C**	178	2 (1.1)	0.652		
** A or B**	341	7 (2.1)			
**Undetectable HIV**					
**RNA**					
** Yes**	282	7 (2.5)	0.138		
** No**	237	2 (0.8)			
**Advanced fibrosis**					
** Yes**	206	5 (2.4)	0.346		
** No**	266	4 (1.5)			
CD4 cell count*^2^					
** <350 cells/mL**	186	3 (1.6)	0.577		
** ≥350 cells/mL**	327	6 (1.8)			
**EPA treatment**					
** Yes**	173	1 (0.6)	0.141	0.432	0.455
** No**	346	8 (2.3)		(0.048–3.915)	
**Starting NRTIs**					
** Yes**	194	4 (2.1)	0.451		
** No**	325	5 (1.5)			
**Starting ATV/r**					
** Yes**	48	5 (10.4)	0.001	6.324	0.014
** No**	471	4 (0.8)		(1.447–27.63)	
**Start of DRV/r or**					
**LPV/r**					
** Yes**	129	0 (0)	0.075	0 (0)	0.996
** No**	390	9 (2.3)			
**Starting raltegravir**					
** Yes**	45	0 (0)	0.439		
** No**	474	9 (1.9)			
**Starting maraviroc**					
** Yes**	24	0 (0)	0.651		
** No**	495	9 (1.8)			

^1^Categorized by median

^2^available in 513 patients

*entered as continuous variable in the multivariate analysis. AOR: adjusted odd´s ratio; EPA: RPV/TDF/FTC; ATV/r: ritonavir-boosted atazanavir; DRV/r: ritonavir-boosted darunavir; LPV/r: ritonavir-boosted lopinavir; NRTI: nucleos(t)ide reverse transcriptase inhibitors

## Discussion

The present study shows that the frequency of grade 3 or 4 TE or grade 4 TBE is very low in HIV/HCV-coinfected patients who receive EPA in the clinical practice. Furthermore, all TE were grade 3 and these hepatotoxic events did not lead to treatment discontinuation.

To date, the little data available on hepatotoxic events in HIV/HCV-coinfected patients who receive RPV is derived from clinical trials. On the one hand, the rates of grade 3 or 4 ALT elevations reported herein are somewhat lower than what was reported in the TMC278-C204 phase II clinical trial, where a frequency of 6% was observed in a population that consisted mainly of patients without HCV coinfection [18]. However, the presence of elevated baseline ALT or AST levels was not taken into account for the definition of TE and therefore, comparisons of these results with those from the present study are difficult to interpret. On the other hand, in a pooled analysis of data derived from the ECHO and the THRIVE clinical trials conducted by Nelson et al [10], TE were among the most frequent adverse events in HCV or hepatitis B virus (HBV)-coinfected subjects receiving RPV in combination with TDF/FTC. In this context, 20.4% of the patients showed grade 2–4 AST elevations and 33.3% of the subjects presented grade 2–3 ALT elevations. However, as it was also the case for TMC278-C204, the number of patients with viral hepatitis was very low in these trials, with only 49 HCV and/or HBV-coinfected patients at baseline and consequently, inferential statistics comparing grade 2–4 TE between patients with and without viral hepatitis were not possible. Furthermore, ART was not applied as a single tablet regimen in these trials and no analysis to distinguish between grade 2 and 3–4 TE was conducted, therefore, the results cannot be compared with the present work. Although results of the STaR and the SPIRIT trials are available, in which EPA was given in a single-tablet regimen in combination with food, unfortunately no specific data on TE or TBE have been published for the coinfected subpopulation [11,12]. Also, over 96% of the participants in these trials did not show HCV coinfection. In contrast, the study described herein includes a high number of HIV-infected subjects with active HCV infection at baseline. Importantly, the frequencies of grade 3 or 4 TE and grade 4 TBE in patients receiving EPA were not only low, they also tended to be under to what is observed for commonly administered ART, as represented by the control group.

Apart from the low frequency of hepatotoxic events, it is important to underline that TE episodes in patients who were given EPA were mild. In this context, no grade 4 TE were observed. Additionally, none of the TE resulted in drug discontinuation. This is an important finding, since patients in a real-life setting may be less motivated to tolerate adverse events and regimens may be switched earlier. Of note, the rate of discontinuations due to liver adverse events or liver-related death was generally low in the EPA group. Finally, the presence of advanced fibrosis or cirrhosis was not associated with an increased risk of grade 3 or 4 TE or grade 4 TBE in patients receiving EPA, which stands in accordance with earlier findings [7,9,19,20]. All this adds up to the safety of EPA in patients with HCV coinfection and is especially important since hepatic safety in cirrhotic patients is crucial for drug selection in this population.

Several studies [7,9,21–24] have assessed the impact of different third agents used in combination with a backbone of two NRTI in subjects with chronic viral hepatitis. These studies found that EFV [7,9] and nevirapine [7] are associated with 5.9–6.6% and 11% of grade 3–4 TE and 2.2–2.6% and 0.8% of grade 4 TBE, PI/r [7,9,21–24] with 8.1–10.5% of grade 3–4 TE and 3.7–15% of grade 4 TBE and raltegravir (RAL) [19] with 9.3% of grade 3–4 TE and 1.9% of grade 4 TBE. Although it is not possible to draw definite conclusions from comparing different studies, given that the cited studies were designed and conducted similar to the present work, EPA appears to be less hepatotoxic than the drugs examined in the other studies.

This study has limitations. First, due to the retrospective character of the study, self-limited hypertransaminasemia may have passed undetected. However, a adequate follow-up was inclusion criteria for this study and it can therefore be ruled out that an episode of severe liver toxicity has gone unnoticed. Second, drugs that were approved after the end of the inclusion period, as dolutegravir and elvitegravir/cobicistat, were not administered to the control group. Still, this study was not aimed to compare specific regimens of the control group and further studies are warranted to clarify this issue.

In conclusion, the frequency of severe liver toxicity in HIV-infected subjects with chronic hepatitis C receiving EPA is very low in the clinical practice. Taken together with the findings that TE episodes in the EPA group were generally mild and did not lead to drug discontinuation, all these data suggest that EPA can be safely used in this particular subpopulation.
